# Combination of Aronia, Red Ginseng, Shiitake Mushroom and Nattokinase Potentiated Insulin Secretion and Reduced Insulin Resistance with Improving Gut Microbiome Dysbiosis in Insulin Deficient Type 2 Diabetic Rats

**DOI:** 10.3390/nu10070948

**Published:** 2018-07-23

**Authors:** Hye Jeong Yang, Min Jung Kim, Dae Young Kwon, Da Sol Kim, Ting Zhang, Chulgyu Ha, Sunmin Park

**Affiliations:** 1 Research Division of Food Functionality, Korean Food Research Institutes, Wanjoo 55365, Korea; yhj@kfri.re.kr (H.J.Y.); kmj@kfri.re.kr (M.J.K.); dykwon@kfri.re.kr (D.Y.K.); 2 Department of Food and Nutrition, Obesity/Diabetes Center, Hoseo University, Asan 31499, Korea; tpfptm14@daum.net (D.S.K.); zhangting92925@gmail.com (T.Z.); 3 Department of Bioprocess Technology, Bio Campus Korea Polytechnic, Nonsan 32943, Korea; hckue@kopo.ac.kr

**Keywords:** aronia, ginseng, mushroom, pancreatectomy, type 2 diabetes, gut microbiome, insulin secretion

## Abstract

The combination of freeze-dried aronia, red ginseng, ultraviolet-irradiated shiitake mushroom and nattokinase (AGM; 3.4:4.1:2.4:0.1) was examined to evaluate its effects on insulin resistance, insulin secretion and the gut microbiome in a non-obese type 2 diabetic animal model. Pancreatectomized (Px) rats were provided high fat diets supplemented with either (1) 0.5 g AGM (AGM-L), (2) 1 g AGM (AGM-H), (3) 1 g dextrin (control), or (4) 1 g dextrin with 120 mg metformin (positive-control) per kg body weight for 12 weeks. AGM (1 g) contained 6.22 mg cyanidin-3-galactose, 2.5 mg ginsenoside Rg3 and 244 mg β-glucan. Px rats had decreased bone mineral density in the lumbar spine and femur and lean body mass in the hip and leg compared to the normal-control and AGM-L and AGM-H prevented the decrease. Visceral fat mass was lower in the control group than the normal-control group and its decrease was smaller with AGM-L and AGM-H. HOMA-IR was lower in descending order of the control, positive-control, AGM-L, AGM-H and normal-control groups. Glucose tolerance deteriorated in the control group and was improved by AGM-L and AGM-H more than in the positive-control group. Glucose tolerance is associated with insulin resistance and insulin secretion. Insulin tolerance indicated insulin resistance was highly impaired in diabetic rats, but it was improved in the ascending order of the positive-control, AGM-L and AGM-H. Insulin secretion capacity, measured by hyperglycemic clamp, was much lower in the control group than the normal-control group and it was improved in the ascending order of the positive-control, AGM-L and AGM-H. Diabetes modulated the composition of the gut microbiome and AGM prevented the modulation of gut microbiome. In conclusion, AGM improved glucose metabolism by potentiating insulin secretion and reducing insulin resistance in insulin deficient type 2 diabetic rats. The improvement of diabetic status alleviated body composition changes and prevented changes of gut microbiome composition.

## 1. Introduction

The prevalence of type 2 diabetes is markedly increasing in Asian countries, including Korea and reached 8.7% of the Asian population by 2014; it is expected to reach 12–5% by 2025 [1]. This is associated with ethnic differences in the etiology of type 2 diabetes [1]. Type 2 diabetes generally develops as a consequence of the imbalance between insulin resistance and insulin secretion [2]. When insulin resistance increases due to obesity, inflammation, oxidative stress, aging, less physical activity, etc., insulin secretion is elevated to overcome insulin resistance and to maintain normoglycemia. Increased inflammation and oxidative stress may accelerate the development of type 2 diabetes especially in Asians. However, Asians are more susceptible to type 2 diabetes under the insulin resistant condition since their insulin secretory capacity and β-cell mass are low [3]. The Westernization of diets mostly elevate insulin resistance in Asians which that is not compensated due to low insulin secretion capacity. Thus, Westernization of diets can increase the prevalence of type 2 diabetes in many cases although obesity is much less in Asian than Caucasians.

Type 2 diabetes is prevented and treated by reducing insulin resistance and increasing insulin capacity in both Asians and non-Asians, although the disease has a somewhat different etiology in Asians than non-Asians. Asians with type 2 diabetes are usually not obese, although they are insulin resistant [3]. Thus, the proper animal model for Asian type 2 diabetes needs to be non-obese, have lower insulin secretion capacity, and higher insulin resistance than the non-diabetic rats. Partially pancreatectomized rats are an optimal model for studying Asian type 2 diabetes [4]. They have about 60% insulin secretion capacity and about 50% of the pancreatic mass of the rats with intact pancreas [4]. The Px rats gradually develop insulin resistance. A high fat diet accelerates the increase of insulin resistance in Px rats [5]. Anti-diabetic interventions for Asians can be examined for improvements in both insulin resistance and insulinotropic activity in Px rats fed with a high fat diet [6].

Previous studies have supported that the gut microbiome produces microbial metabolites including short-chain fatty acids (SCFAs), bile acids (BAs) and lipopolysaccharides (LPS) that modulate host glucose metabolism, mainly in the liver [7]. This is called the gut liver axis. These metabolites directly influence metabolic diseases including type 2 diabetes [7]. Low-grade peripheral inflammation also promotes the development of type 2 diabetes. Patients with a low bacterial α- diversity in the gut microbiome are at greater risk of metabolic diseases than are patients with high α-diversity [8]. Herbs rich in fiber, polyphenols and polysaccharides increase the abundance of the phylum *Bacteroidetes*, and genera *Akkermansia*, *Bifidobacteria*, *Lactobacillus*, *Bacteroides* and *Prevotella* [7]. However, it reduces the number of phylum *Firmicutes* and *Firmicutes/Bacteroidetes* ratio in the intestines. It is well known that some herbal compounds improve glucose metabolism by modulating insulin resistance and insulin secretion. The changes in some of the microbial metabolites from consuming plant compounds are correlated with changes to the gut microbiome that modulate glucose metabolism [9].

*Aronia melanocarpa*, red ginseng and shiitake mushroom have been reported to improve glucose metabolism. Aronia and its anthocyanins have anti-inflammatory and antioxidant properties that improve insulin sensitivity and prevent type 2 diabetes [10]. Red ginseng and ginsenoside Re, Rb1, and Rb2, its active ingredients, have demonstrated an antidiabetic action in in vitro, animal, and clinical studies [11,12,13,14]. Ultraviolet-irradiated shiitake mushroom (Lentinus edodes), polysaccharides, has antioxidant activity and it is a good source for vitamin D (V-D) in humans [5]. The dosage of 100–400 mg shiitake mushroom/kg bw is effective for anti-oxidant properties because it increases the content of the reduced form of glutathione, but at higher dosages it has an adverse effect on immunity [15]. Anti-diabetic effects of V-D are still controversial. V-D may exert anti-diabetic activity by improving insulin secretion and insulin sensitivity [5]. However, some placebo-controlled clinical studies of vitamin D administration have not shown that it improves insulin release and sensitivity [16]. Type 2 diabetes increases the thrombotic risk to develop cardiovascular diseases [17]. Although nattokinase has not been shown shown to possess anti-diabetic activity, it is reported to improve blood flow by inhibiting platelet aggregation and thrombosis to reduce cardiovascular events [18]. The water or ethanol extracts of aronia, red ginseng and shiitake mushroom have been well studied, but the whole foods, including dietary fiber, may be better for inducing changes to the gut microbiome that influence glucose metabolism.

Insulin resistance and β-cell function and mass are associated with increased oxidative stress and inflammation [1,10,19]. Aronia and shiitake mushrooms improve insulin resistance [5,10], red ginseng potentiates β-cell function and mass [11], and nattokinase prevents thrombosis [18]. The combination of aronia, red ginseng, shiitake mushroom and nattokinase, which have anti-oxidative and anti-inflammatory properties, may alleviate type 2 diabetic symptoms and its complications. However, its direct effects on anti-diabetic activity such as insulin resistance and insulin secretion have not been examined. The relationship between anti-diabetic activity and the gut microbiome is also not well characterized. We hypothesized that the combination of freeze-dried aronia, red ginseng, ultraviolet-irradiated shiitake mushroom and nattokinase would prevent or reverse insulin resistance, improve insulin secretion, and help normalize serum glucose levels, due to changes in the gut microbiome. We tested this hypothesis in Px rats, a non-obese type 2 diabetic animal model.

## 2. Materials and Methods

### 2.1. Preparation of the Product and Analysis of Ingredients

Each of aronia, red ginseng, and shiitake mushroom was washed, dried at room temperature, freeze-dried, and powdered. Freeze-dried Aronia, red ginseng, shitake mushroom and nattokinase were mixed with the ratio of (3.4:4.1:2.4:0.1) (Chakreis, AGM) and Chakreis was generously provided by YD Nutraceuticals Ltd. (Yongin-si, Korea). The dosages were determined by considering preliminary studies and previous studies [10,12,13,15,16,18]. The freeze-dried powder mixture was used for the animal study. The mixture was extracted with in distilled water at 95 °C for 12 h and the extracts were centrifuged at 10,000× *g* at 4 °C for 20 min. The supernatants were lyophilized in a freeze-dryer.

For measuring indicative components in the mixture, it was extracted with methanol and lysophilized. The extracts were dissolved in methanol, and a syringe filter was used to remove undissolved contents. The contents of ginsenoside Rg, cyanidin-galactoside, cyanidin-glucoside and cyanidin-arabinoside in the extract were measured were analyzed by high performance liquid chromatography using a Luna C18 column (4.6 mm × 250 mm; ID, 5 µm). The mobile phase solvents were acetonitrile and 0.1% formic acid in water (6:4, vol:vol) with isocratic elution at a flow rate of 1 mL/min, 40 °C in-column temperature, and UV detection at 270 nm. We used ginsenoside Rg, cyanidin-galactose, cyanidin-glucose and cyanidin-arabinoside as standards to quantify the sample.

The β-glucan contents of shitake mushroom were sequentially digested with digestion enzymes by incubating in lower temperature for 2 h. The enzymes used to digest the shitake mushroom were amylase (20 units, pH 6.9) at 20 °C, cellulase (50 units, pH 5.0) at 37 °C, protease (10 units, pH 7.5) at 37 °C, and amyloglucosidase (70 units, pH 4.8) at 60 °C. The digested shiitake mushroom was mixed with 95% ethanol and the mixture was left at 4 °C for 12 h. The mixture was centrifuged at 10,000 rpm for 10 min and water was added into the precipitates. Sulfuric acid was added (1:5) into the diluted precipitate. The mixture was left at room temperature for 20 min and the optical density was measured at 470 nm. Glucose solution was used as a standard.

### 2.2. Animals and Ethics

Eight-week-old male Sprague–Dawley rats (weight, 218 ± 23 g) were housed individually in stainless steel cages in a controlled environment (23 °C; 12-h light/dark cycle). All surgical and experimental procedures were performed according to the guidelines of the Animal Care and Use Review Committee of Hoseo University, Korea (HUACUC-17-57). The rats underwent a 90% pancreatectomy using the Hosokawa technique [20] or received a sham pancreatectomy (sham) under anesthesia induced by intramuscular injection of a mixture of ketamine and xylazine (100 and 10 mg/kg body weight, respectively). The pancreatectomized (Px) rats exhibited characteristics of type 2 diabetes (random glucose levels >180 mg/dL), whereas the sham rats did not [20,21].

### 2.3. Experimental Design

A total of 40 Px rats were assigned randomly to the following four groups, which differed according to diet: (1) 1 g dextrin/kg bw (negative-control) (2) 0.5 g AGM/kg bw, (3) 1 g AGM/kg bw, and (4) 120 mg/kg bw metformin (positive-control). Each group included 10 Px rats. The sham-operated rats were given 1 g dextrin/kg bw for normal-control (*n* = 10). All experimental animals were given free access to water and a high-fat diet containing either the assigned extracts or dextrin for 12 weeks. The high-fat diet was a modified semi-purified AIN-93 formulation for experimental animals [22] that consisted of 42% carbohydrate, 15% protein, and 43% fat. The major carbohydrate, protein, and fat sources were starch and sugar, casein (milk protein), and lard (CJ Co., Seoul, Korea), respectively.

### 2.4. Body Composition Measurement

After calibrating a dual-energy X-ray Absorptiometer (DEXA; Norland pDEXA Sabre; Norland Medical Systems Inc., Fort Atkinson, WI, USA) with a phantom supplied by the manufacturer, the body compositions of the rats were measured at the 7th week of the experimental period. The animals were anesthetized with ketamine and xylazine (100 and 10 mg/kg bw, respectively), and laid in a prone position, with the posterior legs maintained in external rotation with tape. The hip, knee, and ankle articulations were in 90° flexion. Upon the completion of scanning, lean mass was determined in the leg and hip using the DEXA instrument equipped with the appropriate software for the assessment of bone density in small animals [22]. Similarly, the fat mass was measured in the leg and abdominal areas using the DEXA instrument.

### 2.5. Glucose Homeostasis

Overnight fasted serum glucose levels, food intake, and body weights were measured every week. An oral glucose tolerance test (OGTT) was performed at the 7th week in overnight-fasted animals by orally administering 2 g glucose/kg body weight [23]. Blood samples were taken by tail bleeding at 0, 10, 20, 30, 40, 50, 60, 70, 80, 90, and 120 min after glucose loading. The serum insulin levels were determined at 0, 20, 40, 90, and 120 min. The averages of the total areas under the curves for the serum glucose and insulin concentrations were calculated using the trapezoidal rule. At 3 days after OGTT, an intraperitoneal insulin tolerance test (IPITT) was conducted after the food was removed for 6 h. The serum glucose levels were measured every 15 min for 90 min after an intraperitoneal injection of insulin (0.75 U/kg body weight). Serum glucose and insulin levels were analyzed with a Glucose Analyzer II (Beckman Coulter, Palo Alto, CA, USA) and rat Ultrasensitive insulin kit (Crystal Chem, Elk Grove Village, IL, USA), respectively.

### 2.6. Hyperglycemic Clamp and Cerebral Blood Flow

Catheters were surgically implanted into the right carotid artery and left jugular vein in all rats after 7 weeks of treatment and anesthetization with ketamine and xylazine. A hyperglycemic clamp was performed in 10 free-moving and overnight-fasted rats/group after 5–6 days of implantation to determine insulin secretion capacity, as described previously [20,24,25]. During the clamp, glucose was infused to maintain a serum glucose level of 5.5 mM above baseline, and serum insulin level was measured at designated times. After the clamp, the rats were freely provided food and water for 2 days and then deprived of food for 16 h the next day. After anesthesia with a mixture of ketamine and xylazine, the rats were placed in a stereotaxic device with a midline incision of the scalp exposing the periosteum. A small pore was made with a drill in the right lateral ventricle with stereotaxic coordinates: 1.0 mm posterior, 6 mm lateral, 3.7 mm ventral to bregma. A Doppler flow probe was placed on the cerebral vein and blood flow was measured continuously with Laser Doppler Flowmetry (LDF100C-1, BIOPAC Systems, Inc., Goleta, CA, USA) for 10 min.

After measuring cerebral blood flow regular human insulin (5 U/kg body weight; Humulin; Eli Lilly, Indianapolis, IN, USA) was injected through the inferior vena cava. The rats were euthanized by decapitation 10 min later, and tissues were collected rapidly, frozen in liquid nitrogen, and stored at −70 °C for further experiments. Insulin resistance was determined using the homeostasis model assessment estimate of insulin resistance (HOMA-IR) and calculated using the following equation: HOMA-IR = fasting insulin (µIU/mL) × fasting glucose (mM)/22.5. Lipid profiles in the circulation were measured using colorimetry kits from Asan Pharmaceutical (Seoul, Korea).

### 2.7. Immunohistochemistry

Five rats from each group were injected with BrdU (100 µg/kg body weight) after 6 weeks of treatment. The rats were anesthetized intraperitoneally 6 h post-injection with a mixture of ketamine and xylazine, and the brain and pancreas were dissected immediately, perfused with saline and a 4% paraformaldehyde solution (pH 7.2) sequentially, and post-fixed with the same fixative overnight at room temperature [24].

Two serial 5-μm paraffin-embedded tissue sections were selected from the seventh or eighth sections to avoid counting the same islets twice when measuring β-cell area, BrdU incorporation, and apoptosis, were determined as described previously using an immunohistochemistry method [24]. Endocrine β-cells were identified by applying guinea pig anti-insulin and rabbit anti-glucagon antibodies to the sections. Pancreatic β-cell area was measured by examining all non-overlapping images in two insulin-stained sections from each rat at 10× magnification with a Zeiss Axiovert microscope (Carl Zeiss Microimaging, Thornwood, NY, USA). Pancreatic β-cell mass, individual β-cell size, β-cell proliferation by BrdU incorporation, and apoptotic β-cell were measured as described previously [24].

### 2.8. Next Generation Sequencing of the Gut Microbiome

The gut microbiome composition was measured from feces of each rat by analyzing metagenome sequencing using next-generation sequencing [26]. Bacterial DNA was extracted from the samples of each rat using a Power Water DNA Isolation Kit (MoBio, Carlsbad, CA, USA) according to the manufacturer’s instructions. Each library was prepared using polymerase chain reaction (PCR) products according to the GS FLX plus library prep guide. The emPCR, corresponding to clonal amplification of the purified library, was carried out using the GS-FLX plus emPCR Kit (454 Life Sciences, Branford, CT, USA). Libraries were immobilized onto DNA capture beads. The library-beads were added to the amplification mix and oil, and the mixture was vigorously shaken on a Tissue Lyser II (Qiagen, Valencia, CA, USA) to create “micro-reactors” containing both amplification mix and a single bead. The emulsion was dispensed into a 96-well plate and the PCR amplification program was run with 16S universal primers in the FastStart High Fidelity PCR System (Roche, Basel, Switzerland) according to the manufacturer’s recommendations. Sequencing of bacterial DNA in the feces was performed by the Macrogen Ltd. (Seoul, Korea) by a Genome Sequencer FLX plus (454 Life Sciences) as previously reported.

### 2.9. Statistical Analyses

All data are expressed as means ± standard deviations, and all statistical analyses were performed using SAS ver. 9.1 (SAS Institute, Cary, NC, USA). Significant differences among the control, AGM-L, AGM-H, positive-control and normal-control animal groups were identified with one-way analyses of variance. Significant differences in the main effects among the groups were detected using post-hoc Tukey’s tests. A *p*-value <0.05 was considered significant.

## 3. Results

### 3.1. Contents of Anthocyanins and Ginsenoide Rg3

Aronia mainly contained cyanidin-galactoside, but also and cyanidin-glucoside, and cyanidin-arabinoside, whereas red ginseng had mostly ginsenoside Rg3 (2.5 mg/g sample). Shiitake mushroom contained 244 mg β-glucans/g sample (Table 1).

### 3.2. Body Composition

Body weight gains for 11 weeks were higher in the normal-control group than the control group whereas the positive-control and AGM-H increased body weight more than the control group but less than the normal-control (*p* < 0.05). Unlike body weight gain, food intake was not significantly different among the groups. Food efficiency decreased in the control group compared to the normal-control group and its reduction was prevented by AGM-H the most (*p* < 0.05). Epididymal and retroperitoneal fat contents and visceral fat mass, were much lower in the control group than the normal-control group (Table 2). The visceral fat mass was higher in the AGM-L and AGM-H groups than the positive-control group (*p* < 0.05), but it was not significantly different between the control and positive-control groups (Table 2). Thus, less increase of body weight and fat mass might be associated with urinary glucose loss.

**Figure 1:**
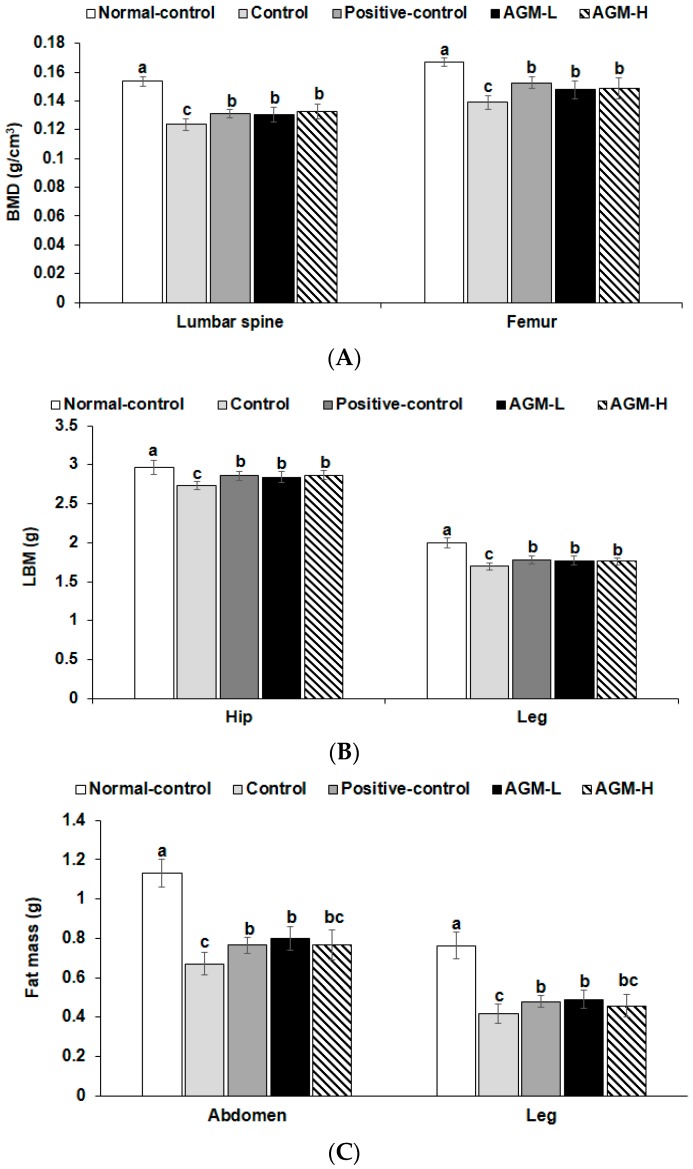
Bone mineral density (BMD), lean body mass (LMB) and fat mass (FM) at the end of experiment. Px rats were fed a high fat diet supplemented with aronia, red ginseng, shiitake mushroom, and nattokinase powders (1) 0.5 g mixture/kg bw/day (AGM-L), (2) 1 g mixture/kg bw/day (AGM-H), (3) 1 g dextrin/kg bw/day (control), or (4) metformin (120 mg/kg body weight; positive-control) for 12 weeks. Sham rats fed the same diet of control. BMD (**A**) in the lumbar spine and femurs, LBM (**B**) of the hip and legs and FM of the abdomen and legs (**C**) were measured by DEXA. Each bar and error bar represents the mean ± SD (*n* = 10 of each group). ^a,b,c^ Different superscripts on the bars represent significant differences at *p* < 0.05.

BMD in the lumbar spine and femur was much lower in the control group than the normal-control group whereas AGM-L and AGM-H prevented the decrease of BMD as much as the positive-control group (*p* < 0.05; Figure 1A). LBM showed a similar pattern of BMD. LBM exhibited a lower value in the control group than the normal-control and AGM-L and AGM-H protected against the decrease of LBM as much as the positive-control group (*p* < 0.05; Figure 1B). Fat mass also showed the similar tendency to LBM (Figure 1C).

### 3.3. Glucose Metabolism

Overnight-fasting serum glucose levels in the control group were higher than those in the normal-control group indicating the diabetic conditions of the control group, and AGM-L and AGM-H decreased the serum glucose levels at fating states (*p* < 0.05; Table 3). Overnight serum insulin levels were lower in the control group than in the normal-control group. As calculated from serum glucose and insulin levels at fasting state, HOMA-IR, an index of insulin resistance, was higher in the control group than in the normal-control (Table 3). AGM-L and positive-control group showed a similar HOMA-IR, and AGM-H was lowered the most (*p* < 0.05). Mean cerebral blood flow was lower in the control than normal-control and it was not significantly different between the control and positive-control. However, AGM-L and AGM-H protected against the decrease in cerebral blood flow in Px rats and the levels in AGM-L were similar to normal-control.

Cerebral blood flow was much lower in the control group than the normal-control group and positive-control did not improve blood flow compared to the control. However, AGM-L and AGM-H prevented the decrease and the level in the AGM-L group was similar to the normal-control group.

**Figure 2:**
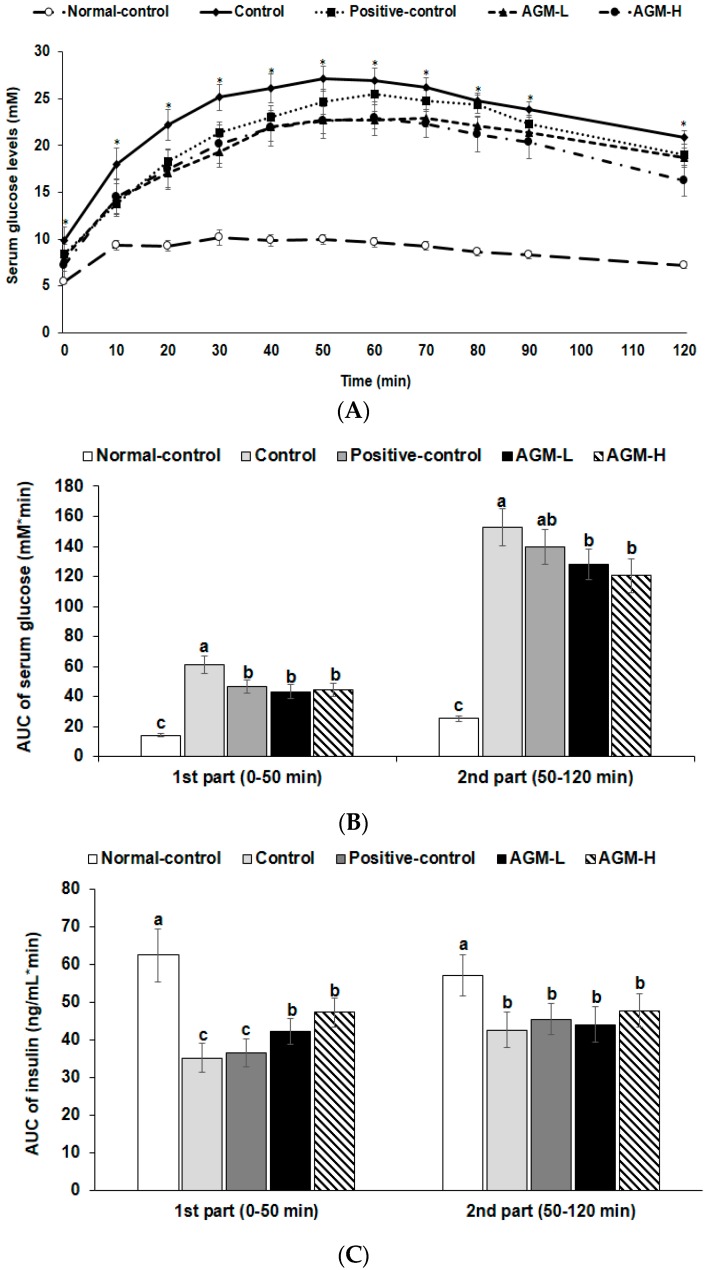
Serum glucose and insulin levels and area under the curve (AUC) of serum glucose and insulin during oral glucose tolerance test (OGTT). Px rats were fed a high fat diet supplemented with aronia, red ginseng, shiitake mushroom, and nattokinase powders (1) 0.5 g mixture/kg bw/day (AGM-L), (2) 1 g mixture/kg bw/day (AGM-H), (3) 1 g dextrin /kg bw/day (control), or (4) metformin (120 mg/kg body weight; positive-control) for 12 weeks. Sham rats fed the same diet of control. Changes of serum glucose levels (**A**) were measured after orally giving 2 g of glucose/kg body weight. The average of the area under the curve (AUC) of glucose (**B**) and insulin (**C**) during the first part (0–40 min) and second part (40–120 min) of OGTT. Each dot and bar and error bar represent the mean ± SD (*n* = 10 of each group). * Significantly different among the groups at each time point at *p* < 0.05. ^a,b,c^ Different superscripts on the bars represent significant differences at *S* < 0.05.

The OGTT revealed that glucose tolerance was highly impaired in the control group compared to the normal-control group and it was improved by metformin in the positive-control group (Figure 2A). AGM-L and AGM-H improved the glucose tolerance better than the positive-control but the improvement did not return it to normal-control group values (*p* < 0.05; Figure 2A). AUC of serum glucose levels during OGTT in the first phase was much higher in the control group than the normal-control group whereas AGM-L and AGM-H decreased the AUC of serum glucose levels (*p* < 0.05; Figure 2B). In the second phase of OGTT, AUC of serum glucose concentrations were much higher in the control group than in the normal-control group and AGM-L prevented the increase (Figure 2B). AGM-H increased the 2nd part of the serum insulin levels in comparison to the control but it was not significantly different (Figure 2C).

### 3.4. Insulin Tolerance

**Figure 3:**
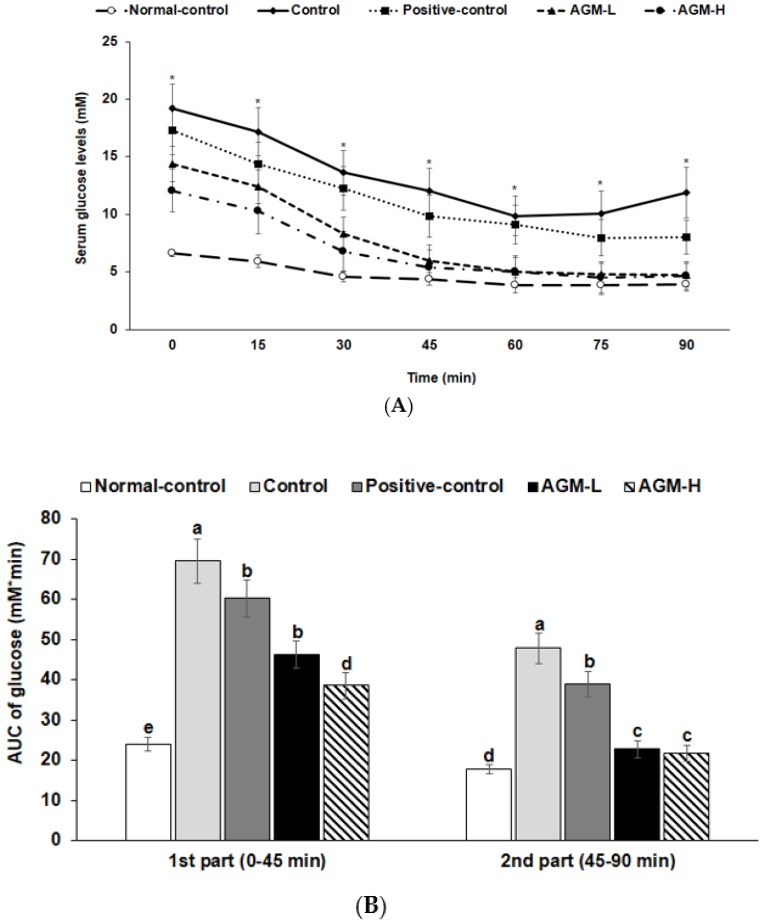
Changes of serum glucose concentrations during the intraperitoneal insulin tolerance test (IPITT). Px rats were fed a high fat diet supplemented with aronia, red ginseng, shiitake mushroom, and nattokinase powders (1) 0.5 g mixture/kg bw/day (AGM-L), (2) 1 g mixture/kg bw/day (AGM-H), (3) 1 g dextrin /kg bw/day (control), or (4) metformin (120 mg/kg body weight; positive-control) for 12 weeks. Sham rats fed the same diet of the control. IPITT was conducted with intraperitoneal injection of 0.75 IU insulin/kg body weight and measured serum glucose concentrations in blood collected from the tail every 15 min for 90 min. Changes of serum glucose levels were measured during IPITT (**A**). The average of the area under the curve (AUC) of glucose (**B**) during the first part (0–45 min) and second part (45–120 min) of IPITT. Each dot and bar and error bar represents the mean ± SD (*n* = 10 of each group). * Significantly different among the groups at each time point at *p* < 0.05. ^a,b,c,d^ Different superscripts on the bars represent significant differences at *p* < 0.05.

At 6 h after food deprivation, serum glucose levels were much higher in the control group than the normal-control group and the levels were lowered in the descending order of the control, positive-control, AGM-L and AGM-H (*p* < 0.05; Figure 3A). After insulin injection, serum glucose levels decreased until 60 min in all groups and the levels were almost maintained in all groups except the control group. The AUC of serum glucose concentrations were much higher in the control than in the normal-control in the 1st and 2nd phase (Figure 3B). AGM-L and AGM-H markedly decreased serum glucose levels during the 1st phase and the levels at the 2nd phase was similar to the normal-control group (*p* < 0.05; Figure 3B). Thus, AGM-L and AGM-H improved insulin tolerance in comparison to the control.

### 3.5. Hyperglycemic Clamp

**Figure 4:**
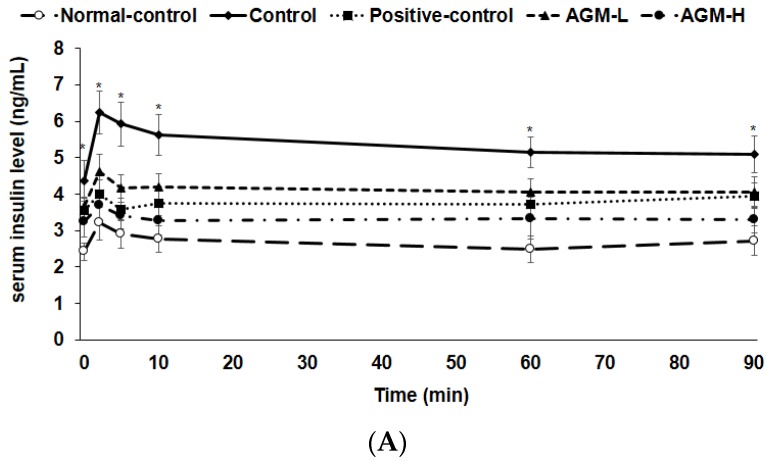

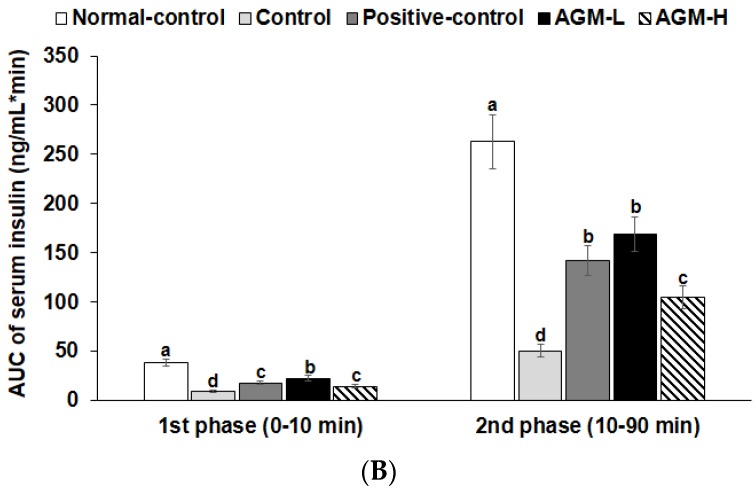
Insulin secretion during hyperglycemic clamp. Px rats were fed a high fat diet supplemented with aronia, red ginseng, shiitake mushroom, and nattokinase powders (1) 0.5 g mixture/kg bw/day (AGM-L), (2) 1 g mixture/kg bw/day (AGM-H), (3) 1 g dextrin /kg bw/day (control), or (4) metformin (120 mg/kg body weight; positive-control) for 12 weeks. Sham rats fed the same diet of the control. Hyperglycemic clamp was conducted in conscious, free moving, and overnight fasted rats to measure glucose-stimulated insulin secretion. As exogenous glucose was infused into jugular vein to make approximately 5.5 mM above overnight fasted serum glucose levels, serum insulin levels were measured at 0, 2, 5, 10, 30, 60, 90 and 120 min (**A**). The average of the area under the curve (AUC) of serum insulin levels (**B**) during the first part (0–10 min) and second part (10–90 min) during hyperglycemic clamp (**B**). Each dot and bar and error bar represents the mean ± SD (*n* = 10 of each group). * Significantly different among the groups at each time point at *p* < 0.05. ^a,b,c,d^ Different superscripts on the bars represent significant differences at *p* < 0.05.

Serum insulin levels were much lower in the control group than normal-control group for 90 min after glucose challenge (*p* < 0.05; Figure 4A). Serum insulin levels exhibited the 1st (0–10 min) and 2nd (60–90 min) phases in all groups. The AUC of 1st and 2nd phases of serum insulin levels increased in AGM-L the most whereas AGM-H elevated the AUC 1st and 2nd phases more than the control but less than the AGM-L (*p* < 0.05; Figure 4B). Glucose infusion rates during hyperglycemic clamp were much lower in the control group than the normal-control group whereas AGM-H prevented the decrease in Px rats, but the levels were less than the normal-control (*p* < 0.05; Table 4). AGM-H showed a higher glucose infusion rates and the levels were higher than the positive-control group (*p* < 0.05). Insulin sensitivity in the hyperglycemic state markedly decreased in the control group compared to the normal-control group and it was higher in the ascending order of control, AGM-H, positive-control and AGM-L (*p* < 0.05; Table 4).

### 3.6. Pancreatic β-cell Mass, Proliferation, and Apoptosis

Pancreatic β-cell area is calculated by the number and individual size of β-cells. The increased number of β-cells improves diabetic status. However, individual β-cell size increases with β-cell hypertrophy that is associated with increased insulin resistance. Pancreatic β-cell area was higher in the control group than the normal-control group, but individual β-cell size was higher in the control group than in the normal-control group (*p* < 0.05; Table 5). AGM-H increased pancreatic β-cell area with smaller individual sized beta-cells, demonstrating that AGM-H increased β-cell number (Table 5). Pancreatic β-cell mass, calculated by multiplying β-cell area by pancreatic weight, was much lower in the control group than the normal-control group. Pancreatic β-cell mass increased in the ascending order of the control, positive-control, AGM-L, and AGM-H (*p* < 0.05; Table 5).

The β-cell number is balanced by β-cell proliferation and β-cell apoptosis. The control rats exhibited a higher β-cell apoptosis than the positive-control rats and AGM-H decreased β-cell apoptosis (*p* < 0.05; Table 5). The β-cell proliferation was lower in the control group than the normal-control group. AGM-H increased the β-cell proliferation (*p* < 0.05; Table 5). Therefore, AGM-H increased β-cell mass by elevating β-cell proliferation and decreasing β-cell apoptosis.

### 3.7. Gut Microbiome

**Figure 5:**
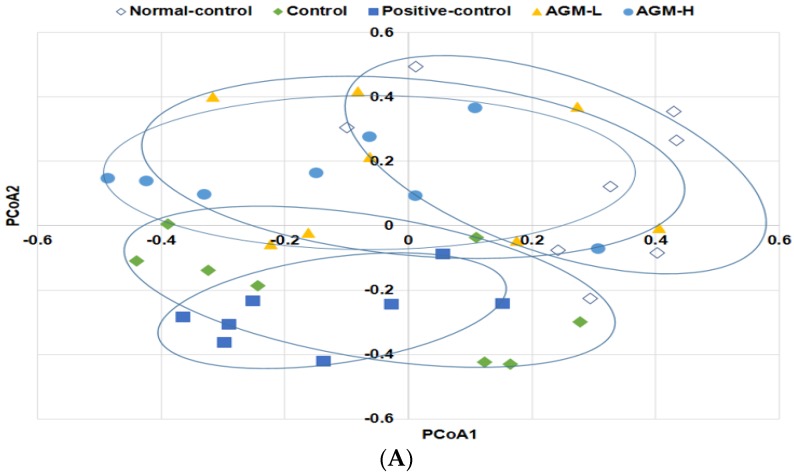

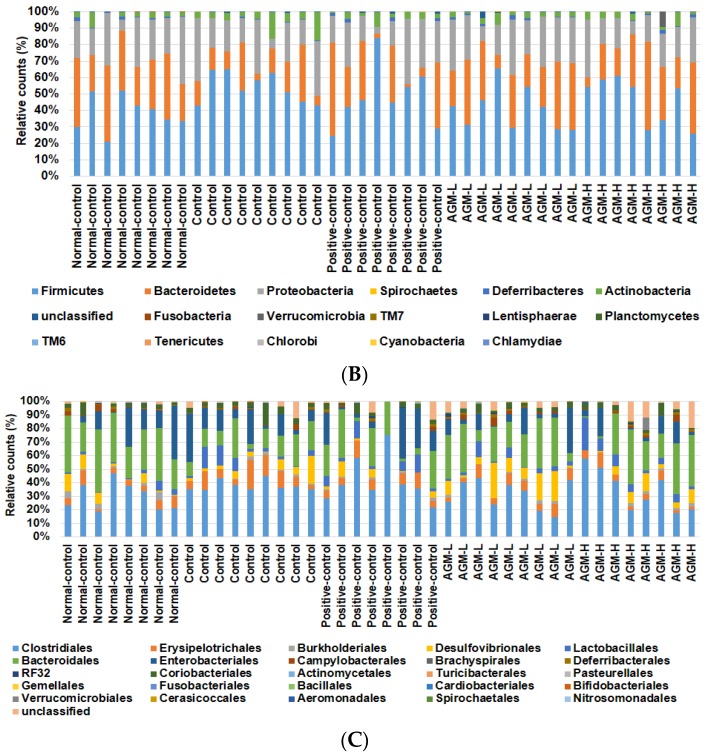
The profiles of gut microbiomes Px rats were fed a high fat diet supplemented with aronia, red ginseng, shiitake mushroom, and nattokinase powders (1) 0.5 g mixture/kg bw/day (AGM-L; *n* = 8), (2) 1 g mixture/kg bw/day (AGM-H; *n* = 8), (3) 1 g dextrin/kg bw/day (control; *n* = 8), or (4) metformin (120 mg/kg body weight; positive-control; *n* = 8) for 12 weeks. Sham rats were fed the same diet as the control (*n* = 8). At the end of experimental periods feces were collected and the bacterial DNA was analyzed. The fecal bacterial community was shown in principal coordinate analysis (PCoA) (**A**). Proportion of taxonomic assignments [Phylum (**B**) and Order (**C**)] for gut microbiomes was analyzed.

Community composition of the gut microbiota was compared with both total and shared operational taxonomic units among the groups by analysis of molecular variance (AMOVA). The AMOVA test revealed significant differences between the fecal bacterial communities among the groups (*p* < 0.01). Principal coordinate analysis (PCoA) illustrates the clustering of gut bacterial community (Figure 5A). Normal-control and control showed a significant separation of gut microbiota. AGM-L and AGM-H also had separate gut microbiota clustering from control but they overlapped with the normal-control. However, positive-control exhibited a similar pattern to that of the control group (Figure 5A). These results indicated that diabetes modulated the composition of gut microbiome and AGM prevented the modulation of gut microbiome.

The bacterial distribution was different among the groups at the phylum and order levels (Figure 5B,C). The major bacteria were *Firmicutes*, *Bacteriodetes*, *Proteobacteria*, *Actinobacteria*, and *Deferribacteres* at the phylum level. The percentage of *Firmicutes* was higher in the control group than the normal-control group and AGM-L and AGM-H reduced its percentage (Figure 5B). In contrast to *Firmicutes*, the percentage of *Bacteroidetes* was lower in the control than the normal-control and it increased with AGM-L and AGM-H (*p* < 0.05; Figure 5B). The percentages of *Proteobacteria*, *Actinobacteria*, and *Deferribacteres* were not altered by diabetic status and AGM supplementation (Figure 5B). The bacteria community was different among the groups in order level more than the phylum level. The percentage of *Bacteroidales* was much lower in the control than the normal-control and it was increased by AGM-L and AGM-H (Figure 5C). The percentages of *Erysipelotrichales* and *Clostridiales* were higher in the control than the normal-control and they were decreased by AGM-L and AGM-H. AGM-L increased the percentage of *Desulfovibrionales* (*p* < 0.05; Figure 5C). Thus, AGM-L modulated gut microbiome to make it similar to the normal-control.

## 4. Discussion

Aronia, red ginseng and ultraviolet-irradiated shiitake mushroom have been reported to influence glucose metabolism. Based on a previous study, freeze-dried aronia, red ginseng, ultraviolet-irradiated shiitake mushroom and nattokinase were mixed at the ratio of 3.4:4.1:2.5:0.1 and the anti-diabetic activity was examined by assessing its efficacy for improving insulin sensitivity and potentiating insulin secretion in non-obese type 2 diabetic rats (Px rats). Px rats fed high fat diets, a well-established model of Asians type 2 diabetes, were used as the animal model for investigating the efficacy of the mixture in the present study. The Px rats had hyperglycemia due to increased insulin resistance and decreased insulin secretion. We used whole food not extracts since the gut microbiome is influenced by dietary fiber in the ingredients and anthocyanins can be easily degraded due to high temperature during extraction. Aronia, red ginseng, ultraviolet-irradiated shiitake mushroom and nattokinase contain different effective components such as anthocyanins, ginsenoside and β-glucan with vitamin D [27,28,29]. The major ingredients are known to be beneficial for alleviating type 2 diabetic symptoms and they are not overlapped between the plants. Thus, the efficacy of the mixture was examined for anti-diabetic activity in the present study.

Hyperglycemia develops when there is insufficient insulin secretion to compensate for insulin resistance. Insulin resistance is due to the impairment of insulin signaling by inflammation, oxidative stress and other factors. Elevated reactive oxygen species (ROS) and proinflammatory cytokines are also associated with impaired insulin signaling and β-cell function, with increasing β-cell apoptosis [30]. The decrease in ROS and proinflammatory cytokines alleviates the diabetic symptoms [29,31]. Hyperglycemia also reduces blood flow, which increases cardiovascular events [32]. The mixture of *Aronia melanocarpa*, red ginseng and mushroom is a good combination for anti-diabetic activity. *Aronia melanocarpa* extracts are rich in anthocyanins that suppress the production of ROS and proinflammatory cytokines [33,34]. Furthermore, aronia extracts prevent hyperglycemia by inhibiting α-glucosidase activity in the small intestines, by their ROS scavenging in humans and animal models [35,36]. Red ginseng can complement the anti-diabetic activity of aronia. Ginsenosides in red ginseng are known to prevent insulin resistance by activating insulin signaling in cells, animals and humans [11,12,13,28]. Red ginseng enhances insulin secretion and increases the pancreatic β-cell mass, which has hypoglycemic effects [14]. A systematic review and meta-analysis of randomized controlled clinical trials demonstrates that ginseng modestly, but significantly, improved fasting blood glucose in non-diabetic and diabetic patients but it does not change hemoglobin A1c and plasma insulin levels [37]. However, Reeds et al. [28] reported that ginseng and ginsenoside Re do not improve insulin sensitivity and β-cell function in obese type 2 diabetic patients. The ginsenosides are not detected in the blood after ginseng and ginsenoside Re treatment due to poor systemic bioavailability of ginsenosides [28]. Shiitake mushroom, which is rich in β-glucans and vitamin D, also has anti-diabetic activities [5,38,39]. β-glucan changes the gut microbiome and may be associated with improving insulin sensitivity. β-glucan mainly reduces insulin resistance to improve glucose metabolism [39]. Koreans have low levels of 25-OH-cholecalciferol in the blood, indicating vitamin D insufficiency that may affect glucose homeostasis with decreasing lean body mass [40,41]. Hyperglycemia increases platelet aggregation and thrombose formation that elevate the susceptibility to cardiovascular events [32]. Nattokinase is reported to suppress thrombosis [17,18]. The present study showed that AGM improved cerebral blood flow to as much as the normal-control. Since serum glucose levels in AGM were higher than the normal-control, the improvement of cerebral blood flow was associated with the factors beyond serum glucose levels. Nattokinase might be involved in the increase of cerebral blood flow in AGM. Therefore, the combination of aronia, red ginseng, shiitake mushroom and nattokinase may have a potent ant-diabetic activity and reduce the diabetic complications such as cardiovascular events. We decided to investigate the anti-diabetic activity of the combination treatment in Px rats. The present study showed that AGM-L and AGM-H improved glucose tolerance by improving insulin sensitivity in a dose-dependent manner, but AGM-L potentiated glucose-stimulated insulin secretion more than AGM-H. Thus, the combination supplementation alleviated the diabetic symptoms in Px rats.

The present study also showed that Px changed the body composition in comparison to the normal-control rats: Body weight and body fat were lower in Px rats compared to the normal-control rats due to increased urinary glucose loss. Fat mass was lower in the Px rats than the normal-control rats and both AGM-L and AGM-H suppressed the decrease in fat mass, but the fat mass of the AGM-L and AGM-H groups was still much lower than that of the normal-control. The suppression of fat mass loss was associated with the reduction of urinary glucose loss. The decrease of fat mass was not prevented as much as the urinary glucose loss. That may be associated with the properties of aronia and ginseng to suppress body fat synthesis and to increase skeletal muscle mass [42,43,44]. Furthermore, Px rats had lower BMD in the lumbar spine and femur and lower LBM in the hip and leg in comparison to the normal-control. These results suggested that the decrease of BMD is associated with lower insulin sensitivity and insulin secretion in diabetic rats. Sufficient insulin release promotes osteoblast activity by binding to insulin receptors in insulin insufficient states [45]. When osteoblasts are activated, osteocalcin is released from the bone and it binds to osteocalcin receptors that are highly expressed in pancreatic β-cells [45]. V-D is weakly correlated with osteocalcin [46] and it may not be involved in osteocalcin activity in insulin signaling. The activation of osterocalcin receptor by binding with osteocalcin promotes β-cell proliferation to improve β-cell function when glucose levels are elevated [47,48]. However, the osteocalcin effect on insulin resistance is still controversial [49,50]. In addition, BMD is associated with peroxisome proliferator-activated receptor (PPAR)-γ activation [41]. Skeletal muscle mass is reduced by decreasing anabolic signaling in the myocytes [51]. Ginsenosides in ginseng are reported to activate the PPAR-γ pathway to improve insulin sensitivity in various tissues [52,53]. The AGM-L inhibited the decrease of BMD and LBM, indicating that the inhibition by AGM was associated with the improvement of insulin sensitivity and potentiating insulin secretion. A potential limitation of the study is stress induced by multiple invasive procedures. However, the stress effects were mitigated by separating invasive procedures by at least one week to allow recovery from the stress. A unique aspect of this study was the use whole herbs instead of their extracts since the gut microbiome plays an important role in host metabolism of energy, glucose and lipids [54]. Dietary fiber works as food for gut microbes which produce short-chain fatty acids [54]. Dietary fibers in lyophilized aronia, red ginseng and shiitake mushroom may improve the composition and richness of the gut microbiome to establish eubiosis. However, another limitation of this study was not to include an AGM extract group in this study. The involvement of the gut microbiome in the development and progression of metabolic diseases is well recognized. Qin et al. has reported that several *Clostridium* species are increased in type 2 diabetes, and butyrate-producing bacteria are decreased [55]. Our preliminary study demonstrated that metabolism of β-glucan produced propionate and butyrate more than dextrin by 3.5 and 2 folds in vitro. However, the effects of dietary fiber in aronia and red ginseng on the microbiome were not examined. The present study also showed that *Clostridales* and *Erysipelorichales*, which are included in the *Fircumicultes.* was higher in the control than the normal-control but AGM decreased them. These changes in the gut microbiome might be associated with modulating the production of propionate and butyrate to influence gut-brain axis [56]. However, metformin treatment, the positive-control, did not alter the *Fircumicutes* although it improved glucose tolerance in the present study. Previous studies have shown that metformin modulates the gut microbiota composition by increasing the growth of some bacteria, such as *Akkermansia muciniphila*, *Escherichia* spp. or *Lactobacillus* and by decreasing the levels of some other bacteria such as *Intestinibacter* [57]. However, metformin treatment has adverse effects such as diarrhea, nausea, heartburn and gas and it may negatively influence the gut microbiome. Further study is needed to elucidate the metformin effect on gut dysbiosis. The present study showed that AGM had a beneficial effect on gut dysbiosis caused by type 2 diabetes: AGM inhibited the increase of *Clostridales* and increased *Bacterioidales* in the type 2 diabetic rats. Therefore, AGM improved glucose metabolism and prevented gut dysbiosis.

## 5. Conclusions

Hyperglycemia caused gut dysbiosis by increasing *Fircumicultes,* and AGM protected against gut dysbiosis. AGM improved glucose metabolism and lipid profiles in insulin insufficient type 2 diabetic rats, with Asian type 2 diabetes. The improved glucose metabolism protected against the decrease in BMD. Thus, AGM may be useful for preventing type 2 diabetes in Asians.

## Figures and Tables

**Table 1:** The contents of ingredients in the mixture.

	Contents (mg/g Powder)
C3-Galactoside	6.22
C3-Glucoside	0.33
C3-Arabinoside	1.53
Total Anthocyanin	8.08
Ginsenoside Rg3	2.5
β-glucan	244

**Table 2:** Energy metabolism and visceral fat mass.

	Normal-Control (*n* = 10)	Control (*n* = 10)	Positive-Control (*n* = 10)	AGM-L (*n* = 10)	AGM-H (*n* = 10)
Body weight gain for 10 week (g)	283 ± 10.5 ^a^	144 ± 10.7 ^b^	169 ± 11.8 ^b^	156 ± 19.1 ^b^	155 ± 13.0 ^b^
Food intake (g/day)	14.4 ± 1.0 ^a^	15.1 ± 0.8 ^a^	15.0 ± 1.0 ^a^	13.4 ± 1.5 ^b^	12.3 ± 1.5 ^b^
Food efficiency	0.30 ± 0.01 ^a^	0.15 ± 0.01 ^d^	0.18 ± 0.12 ^c^	0.19 ± 0.03 ^c^	0.22 ± 0.02 ^b^
Epididymal fat pads (g)	6.7 ± 0.7 ^a^	3.0 ± 0.4 ^c^	2.9 ± 0.3 ^c^	3.8 ± 0.5 ^b^	3.3 ± 0.4 ^c^
Retroperitoneal fat mass (g)	8.1 ± 0.8 ^a^	3.6 ± 0.5 ^c^	3.8 ± 0.5 ^c^	4.3 ± 0.6 ^b,c^	5.0 ± 0.7 ^b^
Visceral fat (g)	14.8 ± 1.6 ^a^	6.7 ± 0.9 ^c^	6.7 ± 0.8 ^c^	8.1 ± 1.0 ^b^	8.3 ± 1.0 ^b^

Food efficiency: daily energy intake/daily weight gain × 100. Values are means ± standard deviation. The test product was the mixture of free-dried aronia, red ginseng, mushroom and nattokinase. Px rats fed a high fat diet supplemented with (1) 0.5 g mixture/kg bw/day (AGM-L), (2) 1 g mixture/kg bw/day (AGM-H), (3) 1 g dextrin/kg bw/day (control), or (4) metformin (120 mg/kg body weight; positive-control) for 12 weeks. Sham-operated rats (normal-control) fed the same diet of control. ^a,b,c,d^ Values on the same row with different superscripts were significantly different at *p* < 0.05.

**Table 3:** Serum glucose and insulin levels at fasting states and insulin resistance.

	Normal-Control (*n* = 10)	Control (*n* = 10)	Positive-Control (*n* = 10)	AGM-L (*n* = 10)	AGM-H (*n* = 10)
Serum glucose at fasting state (mM)	5.4 ± 0.5 ^d^	9.8 ± 0.6 ^a^	8.4 ± 0.6 ^b^	8.1 ± 0.7 ^b^	7.2 ± 0.5 ^c^
Serum insulin at fasting state (ng/mL)	3.78 ± 0.36 ^a^	2.83 ± 0.32 ^c^	3.55 ± 0.35 ^a,b^	3.27 ± 0.34 ^b^	3.23 ± 0.36 ^b^
HOMA-IR	5.4 ± 0.6 ^d^	9.8 ± 1.0 ^a^	8.4 ± 0.9 ^b^	8.1 ± 0.8 ^b^	7.2 ± 0.8 ^c^
Urinary glucose	-	++++	+++	++	+
Mean cerebral blood flow (mm/s)	657 ± 45 ^a^	405 ± 42 ^c^	424 ± 45 ^c^	643 ± 65 ^a^	571 ± 67 ^b^

+, ++, +++, ++++ higher amount of urinary glucose detection with more +. ^–^ No detection of urinary glucose. Values are means ± standard deviation. The test product was the mixture of free-dried aronia, red ginseng, mushroom and nattokinase. Px rats fed a high fat diet supplemented with (1) 0.5 g mixture/kg bw/day (AGM-L), (2) 1 g mixture/kg bw/day (AGM-H), (3) 1 g dextrin/kg bw/day (control), or (4) metformin (120 mg/kg body weight; positive-control) for 12 weeks. Sham-operated rats (normal-control) fed the same diet of control. ^a,b,c,d^ Values on the same row with different superscripts were significantly different at *p* < 0.05.

**Table 4:** Glucose metabolism during hyperglycemic clamp.

	Normal-Control (*n* = 10)	Control (*n* = 10)	Positive-Control (*n* = 10)	AGM-L (*n* = 10)	AGM-H (*n* = 10)
Serum glucose levels at 60 min (mM)	9.8 ± 0.9 ^c^	17.7 ± 1.4 ^a^	14.6 ± 1.7 ^b^	15.1 ± 1.6 ^b^	15.1 ± 1.1 ^b^
Serum glucose levels at 90 min (mM)	9.9 ± 0.9 ^c^	18.3 ± 1.7 ^a^	15.6 ± 1.7 ^b^	15.2 ± 1.3 ^b^	14.6 ± 1.2 ^b^
Serum insulin levels at 2 min (ng/mL)	6.2 ± 0.6 ^a^	3.2 ± 0.5 ^d^	4.0 ± 0.4 ^c^	4.6 ± 0.5 ^b^	3.7 ± 0.4 ^c^
Serum insulin levels at 60 min (ng/mL)	5.1 ± 0.4 ^a^	2.5 ± 0.4 ^d^	3.7 ± 0.3 ^b,c^	4.1 ± 0.5 ^b^	3.3 ± 0.6 ^c^
Glucose infusion rates (umol/kg bw/min)	59.4 ± 3.9 ^a^	26.1 ± 2.8 ^d^	35.6 ± 3.9 ^c^	32.8 ± 4.4 ^c^	43.9 ± 3.9 ^b^
Insulin sensitivity at hyperglycemic state (µmol glucose min−1 100 g−1 per µmol insulin/L)	33.3 ± 3.9 ^a^	19.8 ± 2.4 ^d^	25.8 ± 2.9 ^c^	23.4 ± 2.6 ^c^	28.9 ± 3.3 ^b^

Values are means ± standard deviation (*n* = 10 of each group). The test product was the mixture of free-dried aronia, red ginseng, mushroom and nattokinase. Px rats fed a high fat diet supplemented with (1) 0.5 g mixture/kg bw/day (AGM-L), (2) 1 g mixture/kg bw/day (AGM-H), (3) 1 g dextrin /kg bw/day (control), or (4) metformin (120 mg/kg body weight; positive-control) for 12 weeks. Sham-operated rats (normal-control) fed the same diet of the control. ^a,b,c,d^ Values on the same row with different superscripts were significantly different at *p* < 0.05.

**Table 5:** The modulation of islet morphometry in the pancreas section.

	Normal-Control (*n* = 5)	Control (*n* = 5)	Positive-Control (*n* = 5)	AGM-L (*n* = 5)	AGM-H (*n* = 5)
β-cell area (%)	5.5 ± 0.7 ^c^	6.3 ± 0.8 ^b^	6.8 ± 0.8 ^a,b^	6.9 ± 0.8 ^a,b^	7.6 ± 0.9 ^a^
Individual β-cell size (μm^2^)	185 ± 23 ^c^	239 ± 26 ^a^	209 ± 23 ^b^	206 ± 25 ^b^	189 ± 22 ^b,c^
Absolute β-cell mass (mg)	33.4 ± 2.9 ^a^	17.9 ± 1.9 ^d^	22.4 ± 2.6 ^c^	23.7 ± 2.9 ^c^	28.8 ± 3.5 ^b^
BrdU^+^ cells (% BrdU^+^ cells of islets)	0.72 ± 0.09 ^c^	0.84 ± 0.09 ^b^	0.89 ± 0.11 ^b^	0.90 ± 0.12 ^b^	1.04 ± 0.12 ^a^
Apoptosis (% apoptotic bodies of islets)	0.64 ± 0.07 ^a,b^	0.70 ± 0.09 ^a^	0.66 ± 0.07 ^a,b^	0.65 ± 0.07 ^a,b^	0.59 ± 0.07 ^b^

Values are means ± standard deviation. The test product was the mixture of free-dried aronia, red ginseng, mushroom and nattokinase. Px rats fed a high fat diet supplemented with (1) 0.5 g mixture/kg bw/day (AGM-L), (2) 1 g mixture/kg bw/day (AGM-H), (3) 1 g dextrin /kg bw/day (control), or (4) metformin (120 mg/kg body weight; positive-control) for 12 weeks. Sham-operated rats (normal-control) fed the same diet of the control. ^a,b,c,d^ Values on the same row with different superscripts were significantly different at *p* < 0.05.
